# 2467 PRMT5 is a master epigenetic regulator to promote repair of radiation-induced DNA damage

**DOI:** 10.1017/cts.2018.109

**Published:** 2018-11-21

**Authors:** Jake L. Owens

**Affiliations:** Indiana University School of Medicine

## Abstract

OBJECTIVES/SPECIFIC AIMS: We recently reported that PRMT5 epigenetically activates androgen receptor (AR) in prostate cancer cells. Because targeting AR signaling through androgen deprivation therapy is clinically used as a radiosensitization approach to treat high-risk prostate cancer, our finding raised an exciting possibility that targeting PRMT5 may improve RT for prostate cancer patients. Contrary to our expectation, targeting PRMT5 sensitized both AR expressing and AR negative (AR−) prostate cancer cell lines to radiation. The goal of our study was therefore to determine the role of PRMT5 in repair of IR-induced DSBs and to translate these findings to improving radiation therapy for cancer patients in general (not just prostate cancer patients). METHODS/STUDY POPULATION: The majority of experiments were basic science experiments analyzing PRMT5’s role in the DNA damage response in normal and cancer cell lines. For example, to extend our findings and determine if PRMT5’s role in DSB repair is conserved across multiple cell types, we performed similar experiments in AR− prostate cancer cells, luminal breast cancer cells, glioblastoma cells, and human embryonic kidney cells. To determine the clinical significance of our finding, we also analyzed mRNA expression of PRMT5, AR, and both PRMT5 and AR target genes involved in DSB repair across 43 clinical cancer data sets. RESULTS/ANTICIPATED RESULTS: (1) Targeting PRMT5 sensitizes prostate cancer cells to IR in an AR-independent manner, (2) PRMT5 regulates the repair of IR-induced DSBs in an AR-independent manner, (3) RNA-seq analysis reveals that PRMT5 likely regulates genes involved in the DNA damage response, (4) PRMT5 activates expression of several genes in the DDR including those involved in DSB repair, (5) PRMT5 functions as an epigenetic activator of genes involved in DDR, (6) PRMT5 is required for NHEJ, HR, and G2-Arrest upon IR treatment, (7) Upregulation of PRMT5 correlates with formation and repair of IR-induced DSBs, (8) PRMT5’s role in repair of IR-induced DSBs is conserved in several normal and cancer cell types, and (9) PRMT5 expression correlates with expression of DSB repair proteins in clinical cancer samples. DISCUSSION/SIGNIFICANCE OF IMPACT: In summary, we provide evidence that PRMT5 is a master epigenetic regulator of IR-induced DSB repair through epigenetic activation of multiple target genes involved both HR and NHEJ as well as G2 arrest. Interestingly, the majority of genes regulated by PRMT5 are well-characterized, “core repair proteins” involved in HR (RAD51, BRCA1, BRCA2, RAD51D, and RAD51AP1), NHEJ (NHEJ1, Ku80, XRCC4, and DNAPKcs), and G2 arrest (Cdk1, CDC25C, CCNB2, and WEE1), which may explain why PRMT5 is essential to repair IR-induced DSBs in several cell lines. Although AR may also regulate DSB repair via both HR and NHEJ, several pieces of evidence in our study suggest that PRMT5 also regulates DSB repair independent of AR. First, PRMT5 targeting sensitizes both AR+ and AR− prostate cancer cells to IR. Second, exogenous expression of AR only partially rescues the impairment of IR-induced DSB repair by PRMT5 knockdown. Third, PRMT5 knockdown increases IR-induced DSB in AR− DU145 cells and several other cancer cell lines and normal cells. Fourth, PRMT5 expression correlates positively with the expression of its target genes in multiple human cancer tissues. During preparation of this project, Braun *et al*. reported that PRMT5 post-translationally regulates the splicing out of detained-introns (DI)s of genes to modulate gene expression. However, analysis of their data showed that the majority of DEGs we identified either do not contain DIs or DI splicing was not affected by targeting PRMT5. In addition, Clarke *et al*. reported that PRMT5 participates in the DSB repair choice process and promotes HR through methylation of RUVBL1. It is therefore likely that PRMT5 regulates repair of IR-induced DSB via multiple mechanisms. As PRMT5 is overexpressed in many human cancers and its overexpression correlates with poor prognosis, our findings suggest that increased DSB repair by PRMT5 overexpression in these human cancers may confer survival advantages particularly following DNA damaging treatment. Because targeting DSB repair has been proven to be a valid therapeutic approach for cancer treatment, our findings here also suggest that PRMT5 targeting may be explored as a monotherapy or in combination therapy with RT or chemotherapy for cancer treatment.

